# Fermentation of kefir with traditional freeze-dried starter cultures successfully recreates fresh culture fermented kefir

**DOI:** 10.3389/fmicb.2025.1655390

**Published:** 2025-10-10

**Authors:** Maanasa Mudoor Sooresh, Ashani Jayawickrama, Amaya Silva, Sally Nguyen, Sheri Schmidt, Joseph Sebastian, Shane Carey, James Harynuk, Benjamin P. Willing, Benjamin C. T. Bourrie

**Affiliations:** ^1^Department of Agricultural, Food, and Nutrition Sciences, University of Alberta, Edmonton, AB, Canada; ^2^The Metabolomics Innovation Centre (TMIC), University of Alberta, Edmonton, AB, Canada; ^3^Department of Chemistry, University of Alberta, Edmonton, AB, Canada; ^4^Department of Biological Sciences, MacEwan University, Edmonton, AB, Canada

**Keywords:** fermented foods and beverages, kefir, starter culture fermentation, metabolomics, freeze-drying

## Abstract

**Introduction:**

Interest in fermented foods and their purported health benefits has led to increased scientific research investigating the development of starter cultures which maintain the characteristics of traditional products while allowing for industrial scale production. One such fermented food that is gaining steady attention for industrial production is kefir. To improve the ease of use and maintenance of starter cultures without compromising desirable fermentation characteristics and potential health benefits, this study investigated the impact of freeze-drying a previously described reconstituted kefir consortia with two lyoprotectants trehalose and milk.

**Methods:**

5 bacterial species (*Lentilactobacillus kefiri, Lactobacillus kefiranofaciens, Lactococcus cremoris, Leuconostoc mesenteroides*, and *Acetobacter pasteurianus*) and 4 yeast species (*Saccharomyces cerevisiae, Pichia fermentans, Monosporozyma unispora*, and *Kluyveromyces marxianus*) underwent freeze-drying prior to viability testing and use as starter cultures in kefir fermentations. Completed kefir fermentations were analyzed for pH, microbial composition, volatile compounds, organic acids, and sugar consumption. Freeze-dried starter culture fermentations were compared to kefir made with fresh starter cultures of the same species and pitching rate.

**Results:**

All starter cultures were able to ferment milk to a similar pH, however the freeze-dried cultures prepared with milk took a longer time to complete fermentation. The total bacterial and yeast counts were comparable across the fermentations performed as was the composition of bacteria and yeast present as determined by shotgun metagenomic sequencing. High performance liquid chromatography (HPLC) analysis showed no difference in the levels of lactic acid, acetic acid, ethanol, glucose, and galactose. Additionally, solid-phase microextraction followed by two-dimensional gas chromatography-time-of-flight mass spectrometry (SPME-GC × GC-TOFMS) showed that kefir fermented with freeze-dried starter cultures did not change the volatile profile compared to fresh cultures.

**Conclusion:**

These findings indicate that freeze-dried starter cultures consisting of traditional kefir microorganisms are able to recreate the fresh starter culture version of this product. This provides encouraging evidence for the development of commercially viable starter cultures that are capable of recreating traditional functional fermented foods.

## 1 Introduction

Kefir is a traditionally fermented dairy beverage containing a complex microbial community of yeast and bacteria (Walsh et al., 2016). Typically kefir is made by inoculating milk with kefir grains, an exopolysaccharide matrix containing said microbial community, and allowing fermentation to occur (Louw et al., 2023; Bourrie et al., 2016). While the microbial composition varies slightly among traditional kefirs from different sources, the key players that contribute to the metabolic outcomes include lactic acid bacteria such as *Lactobacillus kefiranofaciens* and *Lentilactobacillus kefiri*, acetic acid bacteria such as *Acetobacter pasteurianus* and yeasts such as *Saccharomyces cerevisiae*, *Pichia fermentans*, *Monosporozyma unispora*, *Kluyveromyces marxianus*, and *Kluyveromyces lactis* (Marsh et al., 2013; Bourrie et al., 2021). The key volatile metabolites produced in kefir fermentations have been traced back to the fungal and bacterial metabolic pathways including acetate esterification, amino acid degradation, carbohydrate metabolism, esterification of fatty acids, fatty acid biotransformation, lipid oxidation, terpene biosynthesis and terpene degradation (Bourrie et al., 2023a,b).

Kefir is associated with a variety of health benefits linked to whole kefir, kefir grains, lactic acid bacteria, yeasts, bacteriocins, organic acids, polysaccharides and other metabolites produced by individual microorganisms or as a result of microbial interactions (Bourrie et al., 2016; Nejati et al., 2020; Walsh et al., 2016). These purported health benefits have led to an increase in the demand for kefir worldwide, with a variety of commercial products rising to meet this demand. However, commercial kefir often significantly varies from traditional kefir in microbial composition and may not always have the same health benefits. For example, a study employed targeted qPCR to check the presence of key microorganisms in traditional kefir grain, traditional kefir and commercial kefir beverage and found that *L. kefiranofaciens* and *Ln. kefiri* along with yeasts such as *Kluyveromyces marxianus, Kazachstania turicensis, Monosporozyma unispora*, and *Dekkera anomala* were not detected in the commercial products (Nejati et al., 2022). Additionally, past work by our group has shown that traditional kefir and products made with traditional kefir microbes are more commonly associated with health benefits than widely available commercial versions (Bourrie et al., 2018, 2021, 2023a,b).

Hallmarks of successful industrial production include process efficiency and consistency of maintaining starer cultures as well as the finished products (Gänzle et al., 2024), while maintaining the positive health impacts. However, for commercial production, it is challenging to work with traditional kefir grains due to inconsistencies and shelf-life concerns (Nejati et al., 2022). Therefore, to devise a method to produce kefir at industrial scale and consistency while maintaining the health benefits of traditional culture, we established a reconstituted consortium of 9 core microorganisms that was able to fully recapitulate the cholesterol lowering benefits associated with traditional grain fermented kefir (Bourrie et al., 2021). The trial also demonstrated the importance of potential microbial interactions in the community as the removal of either the lactobacilli or yeast populations from the fermentation resulted in the amelioration of all health benefits. Furthermore, this product showed greater health benefits in a pilot human clinical trial when compared to a widely available commercial kefir (Bourrie et al., 2023a, 2023b).

To further the development of this novel kefir starter community we employed freeze-drying with the use of lyoprotectants as a technique to standardize the maintenance of the reconstituted kefir consortium (Ge et al., 2024). Freeze drying of microorganisms has been tested for the preservation of probiotic strains as well as for starter cultures for food and beverage fermentations such as kefir, sourdough bread, kombucha and cheese (Chen et al., 2006; Bolla et al., 2011; Fabricio et al., 2022; Ge et al., 2024; Lopes da Silva et al., 2025). While different lyoprotectants can have different effects on the viability of different bacterial and yeast species, and may need to be optimized. This study utilized milk and trehalose as they are readily available and have proven to be effective across a wide range of yeast and bacterial species common to kefir and present in the fermentation community used in the current study (Chen et al., 2006; Bolla et al., 2011; Stefanello et al., 2019). Additionally, these two lyoprotectants allowed us to compare the protective capacity of two inexpensive compounds; a simple sugar, trehalose, and a more complex media comprised of multiple carbohydrates, fats, and proteins in the form of milk. The protective ability of the two agents during freeze drying of a reconstituted kefir microbial community was evaluated through microbial viability, volatile metabolite profile, organic acid production and sugar consumption.

## 2 Materials and methods

### 2.1 Fresh and freeze-dried consortium and kefir fermentation

The kefir consortium contained a mixture of microbes consisting of *A. pasteurianus*, *L. cremoris*, *L. mesenteroides*, *Ln. kefiri*, *L. kefiranofaciens*, *P. fermentans*, *S. cerevisiae*, *M. unispora*, and *K. marxianus*. Bacterial isolates were plated on De Man, Rogosa, and Sharpe (MRS) agar, while yeast isolates were plated on yeast extract, glucose, and chloramphenicol (YEGC) agar. A single colony was picked into MRS or YEGC broth as appropriate and overnight cultures were inoculated in milk at a starting concentration of 10^5^ colony forming units (CFU)/mL of bacteria and 10^4^ CFU/mL of yeast for fresh culture fermentations or used for freeze-drying as described below. The freeze-dried powders were weighed to correspond to the same pitching rate as fresh culture for each isolate (Supplementary Table S2) and this amount was pitched into pasteurized 2% fat milk for fermentation. All kefir fermentations were performed at room temperature for at least 24 h or until pH reached a value below 4.5. The fresh culture, freeze-dried with trehalose (FD Trehalose) and freeze-dried with milk (FD Milk) fermentations reached completion in 24, 24, and 30 h, respectively. Fermentations were conducted in biological triplicate for fresh starter cultures (*n* = 3) and biological sextuplicate for freeze-dried cultures (*n* = 6) with two separate freeze-drying rounds of biological triplicate samples. Discrepancy in sample size ensured that variation between freeze-drying cycles was accounted for as well as because fresh culture fermentations have been previously characterized and found to be consistent across replicates.

### 2.2 Freeze-drying

Cultures for freeze-drying were prepared in the same manner as previously described for fresh culture kefir fermentations. Briefly, overnight cultures of each isolate grown in appropriate medium were centrifuged at 5,000 × g for 10 min at 4⁰C and processed as necessary for each lyoprotectant. For testing trehalose as a lyoprotectant, 1 g of trehalose and 20 mL of fresh broth was added. For testing milk as a lyoprotectant, 20 mL of pasteurized 2% fat milk was added. Samples were then mixed and transferred immediately to an ultra low temperature freezer for storage at −80 °C overnight prior to freeze drying. The yeast and bacterial consortia prepared in milk and trehalose underwent freeze-drying in a VirTis Ultra 35 L freeze dryer at a condenser temperature of −45 °C at an average pressure of 13mTorr for 48 h. The yield of the freeze-dried cultures was determined to be 0.1 g/mL for bacteria and 0.065 g/mL for yeasts. The freeze-dried consortia were stored at 4 °C for 7 days prior to pitching into fermentations.

### 2.3 SPME-GC × GC-TOFMS analysis

Volatile metabolite analysis was performed using a Leco BenchTOF (BT) 4D GC × GC-TOFMS (Leco Instruments, St. Joseph, MI) with a cooled injection System (Gerstel, United States) and a MultiPurpose Sample MPS (Gerstel, United States). 0.5 ± 0.02 g of kefir was placed into a 20 mL headspace vial (VWR, CA) and sealed with magnetic screw caps containing septa (Canadian Life Sciences, CA). Using an automated SPME module (Gerstel, Linthicum, MD), kefir samples were incubated for 5 min at 60 °C then extracted using a three phased SPME (50/30 μm Divinylbenzene/Carboxen/Polydimethylsiloxane, DVB/CAR/PDMS) fiber (SUPELCO, Bellefonte, PA) for 60 min at 60 °C. After extraction, the SPME fiber was desorbed in the inlet at 250 °C for 6 min in splitless mode. First dimension column was a 60 m × 0.25 mm × 0.25 μm Rxi-5SilMS, and the second dimension a 1.3 m × 0.25 mm × 0.25 μm Rtx-200MS (Chromatographic Specialties, Brockville, ON, Canada). Ultra-pure helium (5.0 grade; Praxair Canada Inc., Edmonton) was used as the carrier gas, with a constant flow rate of 2.0 mL/min. Oven temperature started at 80 °C and was held for 3 min then ramped to 240 °C at 3.5 °C/min. The secondary oven and modulator temperature offset were constant at +10 °C and +15 °C, respectively. The modulation period was 2.5 s. Mass spectra were collected at an acquisition rate of 200 Hz over a mass range between 40 and 800 m/z, with an electron impact energy of −70 eV. The detector had a voltage offset of −200 V. The ion source temperature was 200 °C with a transfer line temperature of 250 °C.

Data were processed in LECO ChromaTOF® for BT. Peaks with S/N > 100 were detected. Retention indices were computed based on the elution times of the linear alkanes. All chromatographic peaks were searched against the Version 2.4 NIST database, with a minimum mass spectral similarity of 700 required to assign a putative ID. All sample chromatograms were aligned into a cohesive peak table.

### 2.4 HPLC analysis

Kefir samples were pre-treated with 7% perchloric acid solution in a ratio of 1:1 (v/v) at 4 °C overnight, followed by centrifugation at 10,000 × g for 5 min to remove precipitates. The supernatant was filtered through a 0.22 μm filter and stored at 4 °C prior to analysis. Organic acids and sugars were analyzed using an Agilent 1200 series HPLC system equipped with an Aminex HPX-87H column (300 × 7.8 mm; 9 μm, Bio-rad, United States). Samples were eluted at a flow rate of 0.4 mL/min with 5 mM H_2_SO_4_ as the mobile phase. Quantification was performed on a refractive index (RI) detector and a UV detector (210 nm) using external standards ranging from 0.5 to 40 mM.

### 2.5 Microbial enumeration and pH of kefir

Total bacterial and yeast enumeration was carried out by surface plating of serial 10-fold dilution in phosphate buffered saline on De Man, Rogosa and Sharpe (MRS) agar supplemented with 200 ppm cycloheximide and yeast extract, glucose, and chloramphenicol (YEGC) agar, respectively, at 30 °C. The pH of kefir samples was also monitored using pH meter (Orion 2 Star, Thermo Scientific, Singapore).

### 2.6 DNA extraction and shotgun metagenomic sequencing

One milliliter of kefir sample taken at fermentation completion was pelleted at 10,000 × g for 1 min prior to being resuspended in DNA/RNA shield (Zymo Research, United States). Samples then underwent DNA extraction using the Zymobiomics DNA miniprep kit (Zymo Research, United States) according to manufacturer instructions. DNA was quantified using the Qubit 2.0 fluorometer (Thermo Fisher Scientific) with double-stranded DNA (dsDNA) HS assay kit (Thermo Fisher Scientific). Libraries were prepared using the native barcoding kit 24 (SQK-NBD114.24) from Oxford Nanopore according to manufacturer’s protocol and quantified using the Qubit 2.0 as described above. Fifteen barcoded libraries were pooled in equal concentration prior to adapter ligation and the pooled library was loaded onto a MinION flow cell (R10.4.1) and run on a MinION MK1D with the live super accurate basecalling setting.

### 2.7 Taxonomic profiling of sequence data

Reads were processed using the wf-metagenomics workflow in EPI2ME. Specifically, the Kraken2 workflow was used and aligned to a custom database consisting of reference genomes for each species present in the reconstituted kefir community. Reads with quality scores <10 were removed, along with reads <300 bp. Bracken was used to estimate the relative abundance of each species.

### 2.8 Statistical analysis

The level of significance for all analyses was set at *p* < 0.05. Cell counts, pH and HPLC data was analyzed using Analysis of Variance (ANOVA) with Tukey post-hoc for multiple comparisons. HPLC data plots were created using ggplot2 in R studio 2024.12.1 + 563. The aligned peak table from GC × GC-TOFMS analysis was normalized by the total useful peak area (TUPA). Principal component analysis (PCA) was used to visualize the overall clustering of the three kefir fermentation groups using the TUPA normalized table. The PCA plot was generated using ggplot2. Permutational Multivariate Analysis of Variance using Distance Matrices (ADONIS2) and PERMDISP were used to determine the effect of fermentation type on volatile metabolite and microbial composition and variability. PCoA plot using Bray–Curtis dissimilarity was generated using the phyloseq and ggplot2 R packages.

## 3 Results

### 3.1 Bacterial starter cultures had higher levels of survival following freeze-drying than yeasts

Bacterial and yeast viability for both FD Milk and FD Trehalose groups are reported in Table 1. Bacteria had viability levels ranging between 100.26 and 96.45% with no differences observed between the two lyoprotectants. While yeasts had lower levels of viability (84.18–86.63%), there was similarly no difference between the two lyoprotectants (Table 1).

**Table 1:** Viability percentage of individual bacterial and yeast species following freeze drying with milk (FD Milk) and trehalose (FD Trehalose).

Species	Fresh vs. FD milk (viability %)	Fresh vs. FD trehalose (viability %)
*Lentilactobacillus kefiri*	99.71 ± 0.72	100.26 ± 0.79
*Lactobacillus kefiranofaciens*	97.60 ± 1.20	97.65 ± 0.73
*Lactococcus cremoris*	96.45 ± 1.86	97.65 ± 1.26
*Leuconostoc mesenteroides*	97.25 ± 0.81	96.45 ± 1.08
*Acetobacter pasteurianus*	97.31 ± 0.94	96.89 ± 0.85
*Saccharomyces cerevisiae*	85.87 ± 1.28	84.18 ± 1.42
*Pichia fermentans*	85.67 ± 1.01	84.78 ± 1.91
*Monosporozyma unispora*	86.63 ± 1.43	84.78 ± 1.91
*Kluyveromyces marxianus*	85.65 ± 0.80	84.63 ± 1.33

Data are shown as means ± standard deviation. *n* = 6.

### 3.2 FD Milk kefir differed in pH but not cell count when compared to fresh culture kefir and FD trehalose

Total bacteria and yeast were measured at the end of fermentation. Both the freeze-dried starter culture kefirs did not differ in total bacteria or total yeast when compared to kefir produced with fresh starter cultures (Table 2). When comparing pH values of the freeze-dried and fresh culture kefir at 24 h, the pH of milk was significantly higher than fresh and trehalose, however the final pH of the FD Milk fermentations did not differ from either fresh or FD Trehalose (Table 2).

**Table 2:** Microbial cell counts (Log CFU/mL) and pH of fermented kefir with fresh culture, culture freeze-dried with milk and culture freeze-dried with trehalose.

Starter culture	Total bacteria (Log CFU/mL)	Total yeast (Log CFU/mL)	pH at 24 h	pH at completion
Fresh	9.14 ± 0.04	5.94 ± 0.04^a^	4.35 ± 0.03^b^	4.35 ± 0.03
FD milk	9.03 ± 0.06	5.36 ± 0.18^b^	4.76 ± 0.03^a^	4.42 ± 0.03
FD trehalose	9.16 ± 0.04	5.53 ± 0.25^b^	4.37 ± 0.02^b^	4.37 ± 0.02

Data are shown as means ± standard deviations of the 6 biological repeats. Means that do not share a letter are significantly different (*n* = 6).

### 3.3 Kefir starter culture treatment has a minor impact on volatile metabolite profiles but not organic acid and sugar profiles

**Figure 1:**
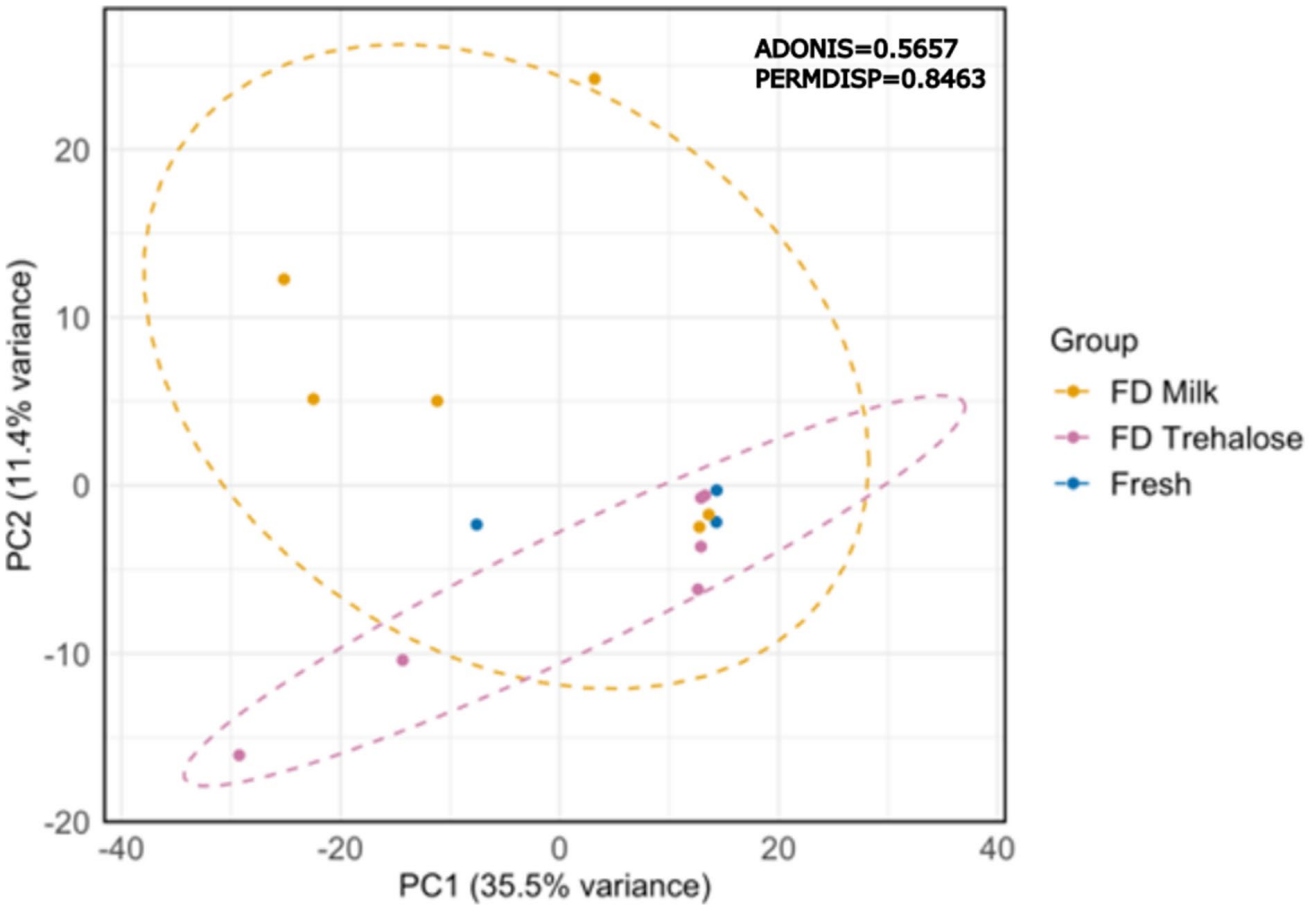
PCA of volatile metabolites in kefir after fermentation prepared with fresh (*n* = 3) or freeze-dried starter cultures (*n* = 6); PERMDISP *p* = 0.8463; PERMANOVA *p* = 0.5657.

**Figure 2:**
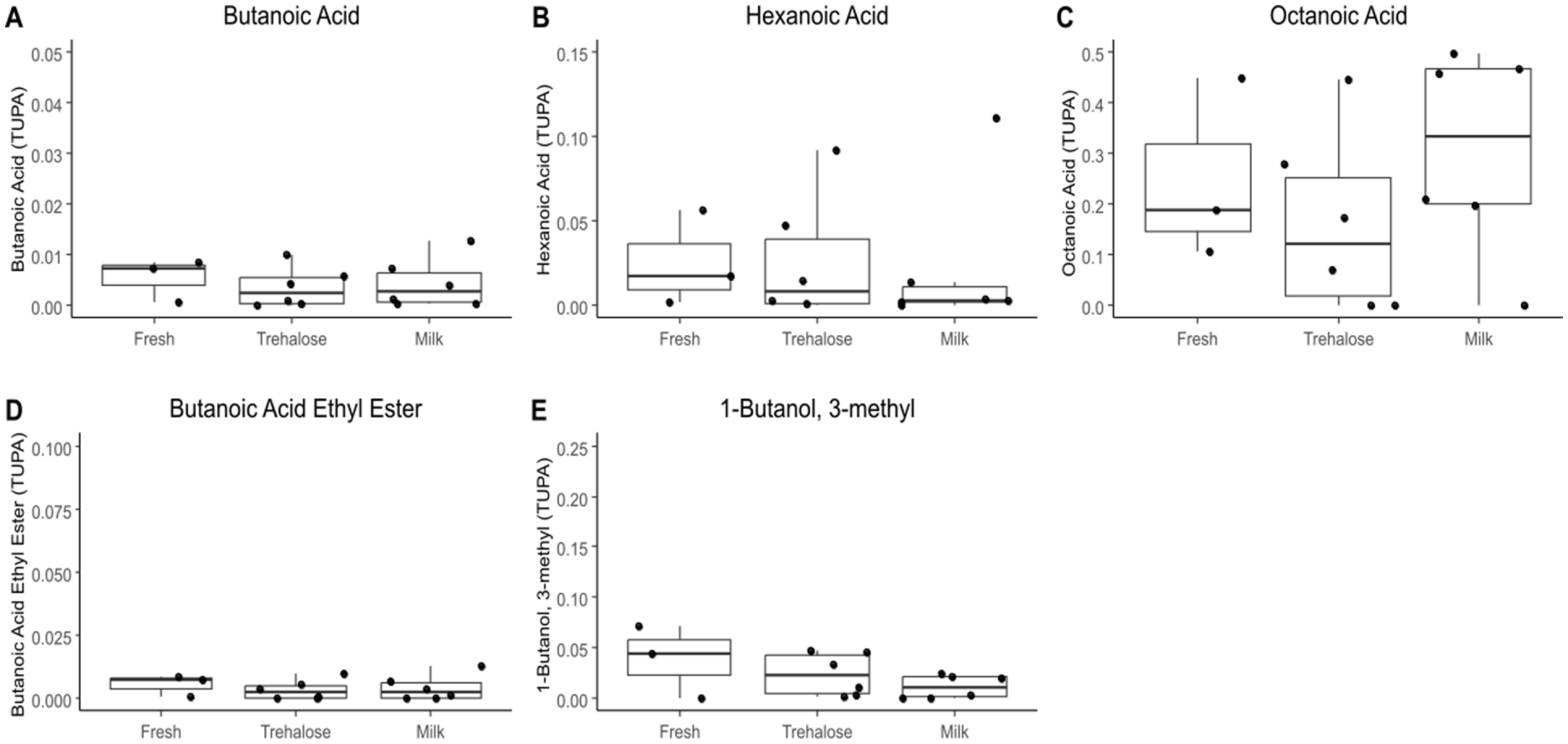
Boxplots showing total useful peak area (TUPA) of butanoic acid **(A)**, hexanoic acid **(B)**, octanoic acid **(C)**, butanoic acid ethyl ester **(D)**, and butanal-3-methyl **(E)** between kefir fermentations performed with fresh culture, freeze-dried cultures with milk and freeze-dried cultures with trehalose as measured by GCxGC-TOFMS. Data are expressed as mean values with their standard errors (*n* = 3–6).

**Figure 3:**
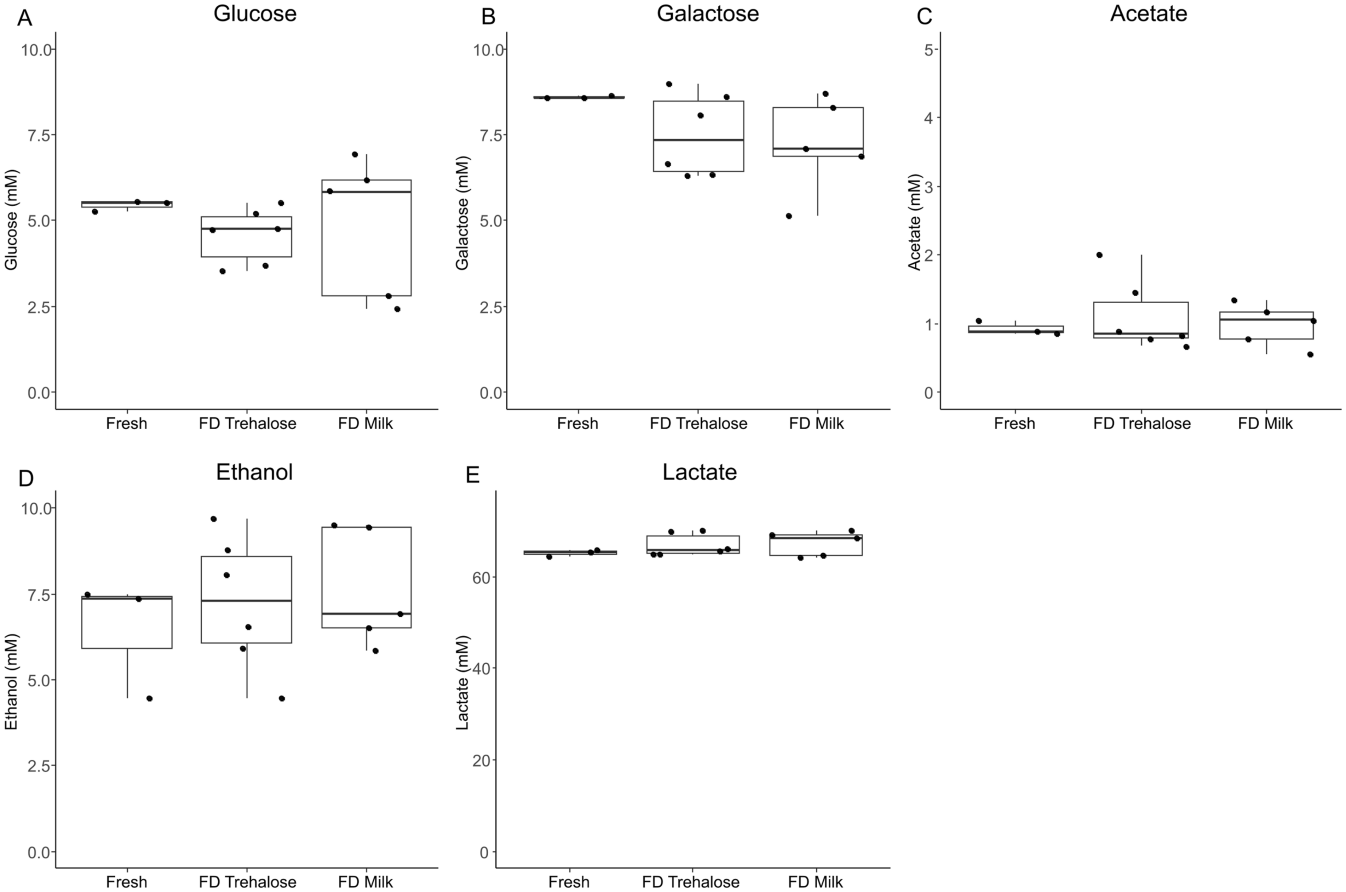
Boxplots showing concentration (mM) levels of glucose **(A)**, galactose **(B)**, acetic acid **(C)**, lactic acid **(D)**, and ethanol **(E)** between kefir fermentations performed with fresh culture, freeze-dried cultures with milk and freeze-dried cultures with trehalose as measured by HPLC. Data are expressed as mean values with their standard errors (*n* = 3–6).

PCA analysis of kefir metabolite profiles showed that there was no clustering based on starter culture treatment (Figure 1). Both PERMDISP and PERMANOVA did not result in any significant findings when comparing fresh and freeze-dried starter culture kefirs (*p* = 0.8463 and 0.5657 respectively), indicating that there was no significant differences in the variability or overall metabolite profile for each preparation. A total of 774 compounds were detected by SPME-GC × GC-TOFMS, of which 72% (556 compounds) were tentatively identifiable by name based on mass and retention index. Identified compounds included organic acids, esters, and aldehydes including butanoic, octanoic, and hexanoic acid, butanoic acid ethyl ester, and butanal-3-methyl; each of which are key flavor metabolites in kefir fermentation and have been previously identified in the fresh culture fermentations by our group (Figure 2). Further analysis identified no compounds as being significantly different in TUPA between groups when a false discovery adjusted *p* value of <0.10 was applied. HPLC analysis revealed that there was no difference in the concentration of any of glucose, galactose, acetic acid, lactic acid, or ethanol when comparing the three kefir preparations (Figure 3).

### 3.4 Shallow metagenomic sequencing reveals microbial communities remain unchanged between fresh and freeze-dried starter culture kefirs

**Figure 4:**
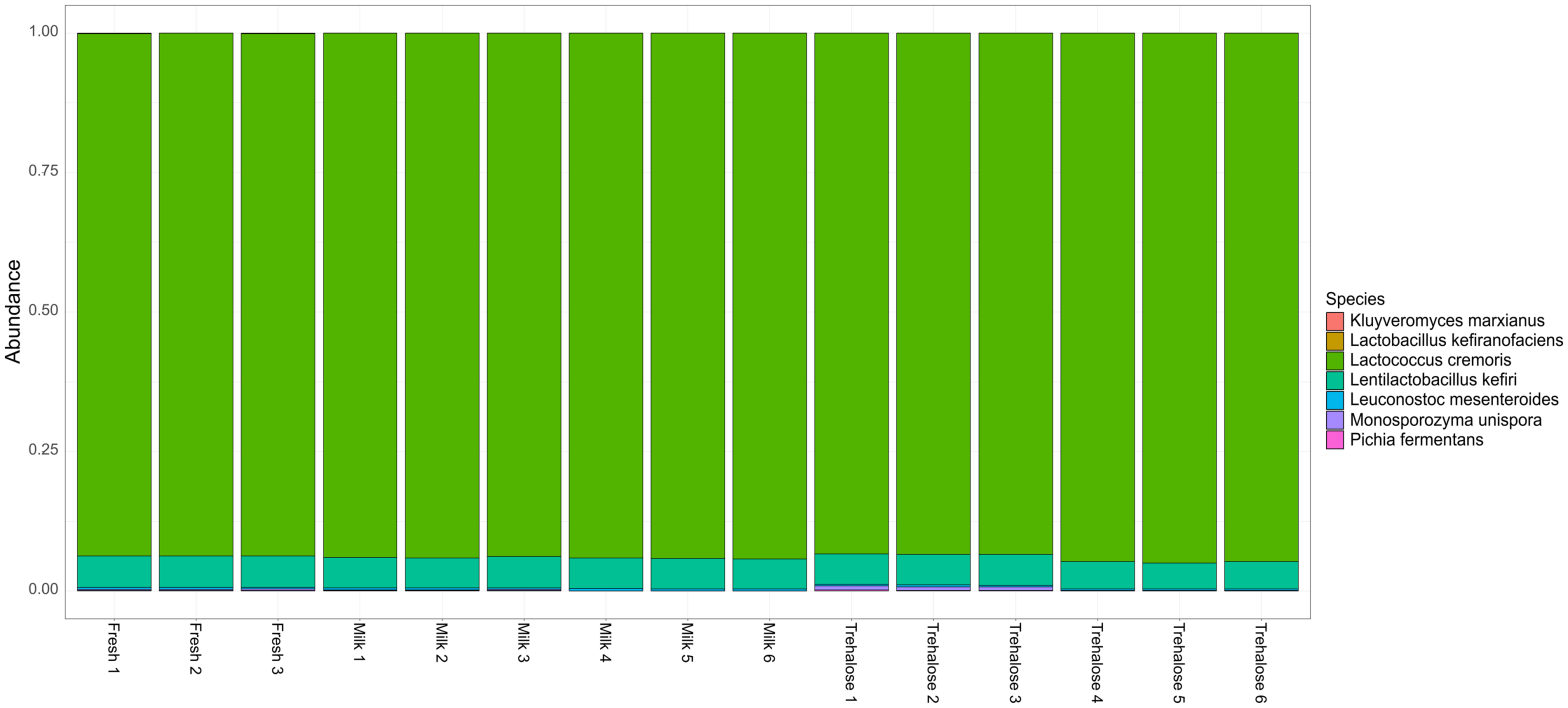
Stacked bar charts representing relative abundance at the species level for each kefir sample at the completion of fermentation.

The number of reads per sample following quality filtering was 55,573 ± 8,140 with an average read length of 3.8 kb. Taxonomic analysis of the overall composition and variance of microbial communities present in the kefir at completion of fermentation revealed no differences between groups (ADONIS = 0.284, PERMDISP = 0.285; Supplementary Figure S3). Samples were universally dominated by *L. cremoris*, representing between 93.4 and 95.0% in all samples with the second most abundant microbe being *Ln kefiri* (4.6–5.7%; Figure 4). While not present at above 1% relative abundance, *L mesenteroides.*, *M. unispora*, *K. marxianus*, and *P. fermentans* were also identified in the majority of samples, while *L. kefiranofaciens* was only identified in a single sample. Additionally, LEfSe analysis did not reveal any differentially abundant OTUs between groups (adjusted *p* > 0.05).

## 4 Discussion

This study sought to determine how trehalose and milk, two common lyoprotectants, influenced the viability and metabolic activity of freeze-dried kefir starter cultures comprising the core bacteria and yeast species found in traditional kefir. In particular, microbial composition was profiled using shallow metagenomic sequencing, and HPLC, and GCxGC-TOFMS used to determine volatile metabolites, organic acids, and simple sugars in kefir produced with fresh and freeze-dried starter cultures. This study is one of the first to examine how freeze drying impacts the fermentative ability of a reconstituted kefir consortia consisting of 9 different species of microorganisms with established health benefits in both human and animal trials (Conde-Islas et al., 2019).

Freeze drying with lyoprotectants has been employed in many different studies pertaining to yeast and bacteria that are industrially relevant as a means of preservation and standardization of starter cultures while maintaining high viability (Stefanello et al., 2019; Conde-Islas et al., 2019). In our study, we tested the use of trehalose and milk as lyoprotectants during freeze drying of 5 bacterial and 4 yeast species that have previously been used as a reconstituted starter community for kefir fermentation (Bourrie et al., 2021, 2022, 2023a,b). Indeed, we found that both trehalose and milk were able to maintain high levels of viability in bacteria and yeasts, although the viability in yeast species was lower. While the yeast had lower viability levels (between 85 and 86%), this is in line with previous reports with gram positive and gram negative bacteria generally having significantly higher rates of survival following freeze drying (Miyamoto-Shinohara et al., 2000; Navarta et al., 2020). For instance, a study showed that in the presence of trehalose and reconstituted skim milk, the cell viability in *Limosilactobacillus reuteri* was higher when compared to using phosphate buffer saline as a negative control, indicating that the protectants may positively influence cell viability (Li et al., 2011). Another enzyme important for both kefir fermentation protection during freeze drying is β-galactosidase, whose activity has been shown to increase significantly in the presence of different cryo and lyoprotectants (Yuan et al., 2025). It is also possible that the production of exopolysaccharide by the lactobacilli present plays a role in their high level of viability and survival of the freeze-drying process as increased polysaccharide production has been correlated with increased viability in a range of lactic acid bacteria (Nguyen et al., 2014, 2022).

Following confirmation of microbial viability, we examined the ability of the freeze-dried starter cultures to ferment milk when compared to fresh cultures. Viability measures were used as a measure to adjust the pitching rate of freeze-dried cultures to ensure equal numbers of microbes were pitched into each fermentation. While we found that freeze-dried starter cultures prepared with both lyoprotectants were able to successfully ferment milk, there were some differences in the fermentation profiles. Specifically, FD Milk starters required longer to reach a pH value of <4.6 which is the critical control point for kefir fermentation. This slower fermentation rate may indicate that trehalose is the preferable lyoprotectant for this particular set of microorganisms and fermentation. This difference in fermentation time may indicate a slight delay in the metabolism of the FD milk organisms at the beginning of fermentation. Interestingly, while there were differences in the fermentation time of the different kefir fermentations, we found that freeze drying the cultures with lyoprotectants did not significantly impact the total bacterial and yeast cell counts at the end of kefir fermentation. Additionally, shallow metagenomic sequencing showed that the microbial composition of the finished kefir was the same for all groups. Specifically, we found that the finished kefir was dominated by *Lactococcus cremoris* (~95% abundance), with *Ln. kefiri* also present at ~5%. Other organisms of the consortia including *L. mesenteroides*, *M. unispora*, *K. marxianus*, and *P. fermentans* were also detected although they were all present at ≤0.5%. While the level of *Lactococcus* in the finished fermentations was high, this is not uncommon among traditional kefir fermented using kefir grains (Walsh et al., 2023). Together, these findings indicate that while fermentation time may be affected by the use and type of freeze-dried starter cultures, there is little difference in the composition of the final product. However, future work should examine the temporal variability of these fermentations to gain a better understanding of exactly how these starter culture preparations impact the entirety of the fermentation.

Delayed metabolic capabilities may be induced because of re-acclimatizing from a stressful environment. Other markers include the presence and expression of stress-related genes. Trehalose has been widely acknowledged for helping yeast cells in ethanol stress and cold stress (Stewart, 2010; Gibney et al., 2015; Tapia et al., 2015). It is interesting to note here that some yeast species, in addition to being able to synthesize trehalose, possess a trehalose transport protein AGT1 that aids in the transport of extracellular trehalose together with the presence of TDH3 promoter (Chen and Gibney, 2022). In addition, Chen and Gibney noted that the protective effects of trehalose were most pronounced when freeze dried in a concentrated pellet rather than in liquid media. These factors provide an opening to understanding how the freeze-dried bacteria and yeast are able to maintain their viability and metabolic functions compared with fresh cultures. Whole genome sequencing and gene expression analysis can reveal key information regarding the presence of genes participating in freeze drying stress resistance of industrially relevant and important yeast and bacterial species, leading to starter culture improvement.

Following fermentation, HPLC analysis was conducted on the final kefir products to determine whether freeze drying of starter cultures impacted the metabolite profiles present. We found that there were no significant differences between the fresh starter cultures and either of the freeze-dried cultures in any of the measured organic acids or sugars. Additionally, volatile metabolite profiling found that there were no significantly different compounds when comparing fresh culture kefir to either the trehalose or milk freeze-dried starter cultures. Together, these findings indicate that the overall metabolic profile of the microbial community remains unchanged following freeze drying. The key volatiles detected were also observed in our previous study using the same microbial consortium as the present study. Elevated levels of the key volatiles, butanoic acid, butanoic acid ethyl ester, hexanoic acid and octanoic acid, were observed for kefir fermentation performed in the presence of *Ln. kefiri* and *L. kefiranofaciens* (Bourrie et al., 2023a). Thus, signifying the importance of these kefir-associated lactic acid bacteria (LAB) in the production of these key volatiles during kefir fermentation. While the two lactobacilli were not identified at high relative abundance at the completion of fermentation, it is possible that these organisms are more active during earlier stages of fermentation leading to the production of these volatile fatty acids and esters. It has also been shown that *L. cremoris* is capable of producing butanoic acid ethyl ester in dairy fermentations (Liu et al., 1998). Past work has shown that the production of these fatty acids during kefir fermentation may be attributed to lipid metabolism by a variety of LABs during fermentation (Walsh et al., 2016). These results are encouraging for the potential implementation of freeze drying as a strategy to increase the potential shelf-life and accessibility of this product.

Furthermore, the key metabolites belonging to short-chain fatty acid (SCFA) and medium-chain fatty acid (MCFA) produced during kefir fermentation have been associated with health benefits. The health benefits of SCFAs, acetic acid, lactic acid and butanoic acid, detected in the current study include regulation of immunity, maintaining the structural integrity of intestinal mucosa and modulation of host immune response (Chen et al., 2024). Furthermore, the MCFAs, hexanoic acid and octanoic acid, have also been reported to afford profound health benefits. For instance, hexanoic acid was observed to improve the expression levels of genes associated with gluconeogenesis and improve insulin sensitivity of mice provided with high-fat diet (Ikeda et al., 2025). Additionally, hexanoic acid and octanoic acid have shown to favor lipid catabolism as well as maintain an optimal insulin sensitivity during *in-vitro* study using HepG2 human hepatocellular carcinoma cells (Rial et al., 2018). The positive health benefits of MCFAs, especially octanoic acid, was further demonstrated in an *in-vivo* study with octanoic acid-enriched diet. The authors observed that the enrichment resulted in improved endurance as a result of improved mitochondrial biogenesis, which lead to an increase in skeletal muscle oxidative capacity (Charlot et al., 2022). These observations further support the health benefits of the kefir produced using freeze-dried starter culture.

Overall, the results of this experiment provide strong initial evidence that the pitched kefir product used in past studies to provide metabolic health benefits such as lowering plasma cholesterol levels can be successfully recreated using freeze-dried starter cultures. The use of both trehalose and milk as lyoprotectants resulted in high viability and successful fermentations with no discernable differences in microbial composition or metabolite profile when compared to fresh culture fermented kefir. It should be noted that while these results are encouraging, there is still much to understand about the impact of freeze drying on these starter cultures. While the microbial cell counts and composition were the same across all three kefir fermentations, these were analyzed only at the completion of fermentation. While this timepoint would align with the state of the product at its time of consumption, there is still much to learn regarding the dynamics of these fermentations, particularly during earlier stages. Future trials should consider temporally sampling the fermentation to assess fermentation dynamics across its entirety. Additionally, while metabolite profiles did not appear to be different based on the analysis carried out, it is possible that there were differences in metabolites that were not analyzed such as peptides. Peptides are particularly important in dairy fermentations as they can provide flavor and other sensory characteristics as well as influence health benefits through the presence of bioactive peptides (Gobbetti et al., 2002; Fitzgerald and Murray, 2006; Gobbi et al., 2019). In addition, future work evaluating the sensorial attributes such as taste, odor, aroma, texture, flavor, appearance and overall acceptability which are deemed important for kefir fermentation must be performed to determine the potential for commercialization (Irigoyen et al., 2005; Kök-Taş et al., 2013). Future studies should also investigate the potentially variability of both fresh and freeze-dried starter cultures across a large number of fermentations to ensure that product consistency remains. Another important factor in potential commercialization of this product would be shelf-life. While freeze-drying often results in increased shelf-life, the specific length of time should be investigated under multiple conditions to ensure consistency across fermentations. Overall, this study provides encouraging evidence that freeze drying is a valid strategy for the standardization of starter cultures associated with traditional food fermentations and may allow for increased access to fermented products with potentially beneficial health effect.

## Data Availability

The raw data supporting the conclusions of this article will be made available by the authors, without undue reservation.
