# Supplementary material for: Fermentation of kefir with traditional freeze-dried starter cultures successfully recreates fresh culture fermented kefir
Source: Front Microbiol. 2025 Oct 10;16:1655390. doi: 10.3389/fmicb.2025.1655390 (PMC12549565; doi:10.3389/fmicb.2025.1655390)
Supplement: Supplementary file 1 [file Data_sheet_1.docx]

**Supplemental Materials for “Fermentation of Kefir with Traditional Freeze-Dried Starter Cultures Successfully Recreates Fresh Culture Fermented Kefir”**

**Supplemental Tables**

Supplemental Table 1. Pitching rates (g/L) for freeze-dried starter culture kefir fermentations. Data are shown as means ± standard deviations of 6 biological repeats. Significantly different pitching rates are indicated by a *.

| **Organism** | **FD Trehalose Pitching Rate** | **FD Milk Pitching Rate** |
| --- | --- | --- |
| ***L. kefiri*** | 0.045±0.020 | 0.050±0.012 |
| ***L. kefiranofaciens*** | 0.035±0.025 | 0.033±0.019 |
| ***L. cremoris*** | 0.025±0.012 | 0.038±0.027 |
| ***L. mesenteroides*** | 0.034±0.024 | 0.026±0.010 |
| ***A. pasteurianus*** | 0.034±0.026 | 0.029±0.013 |
| ***S. cerevisiae*** | 0.300±0.115 | 0.217±0.069 |
| ***P. fermentans*** | 0.276±0.110 | 0.236±0.095 |
| ***M. unispora*** | 0.271±0.113 | 0.211±0.085 |
| ***K. marxianus*** | 0.284±0.095 | 0.241±0.096 |

**Supplemental Figures**

**
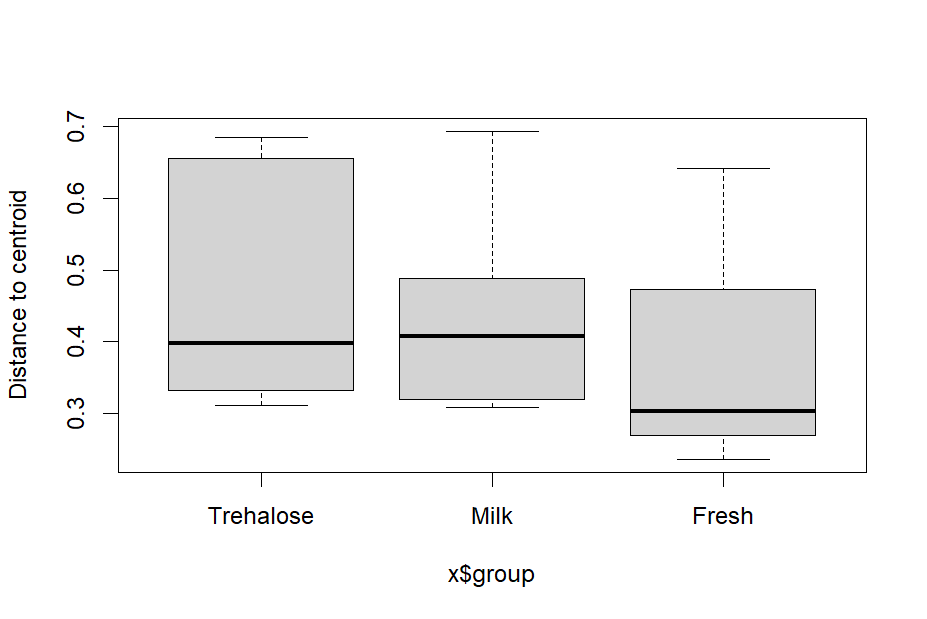
**

Supplemental Figure 1. Distance to the centroid as measured by betadisper analysis for each fermentation group.


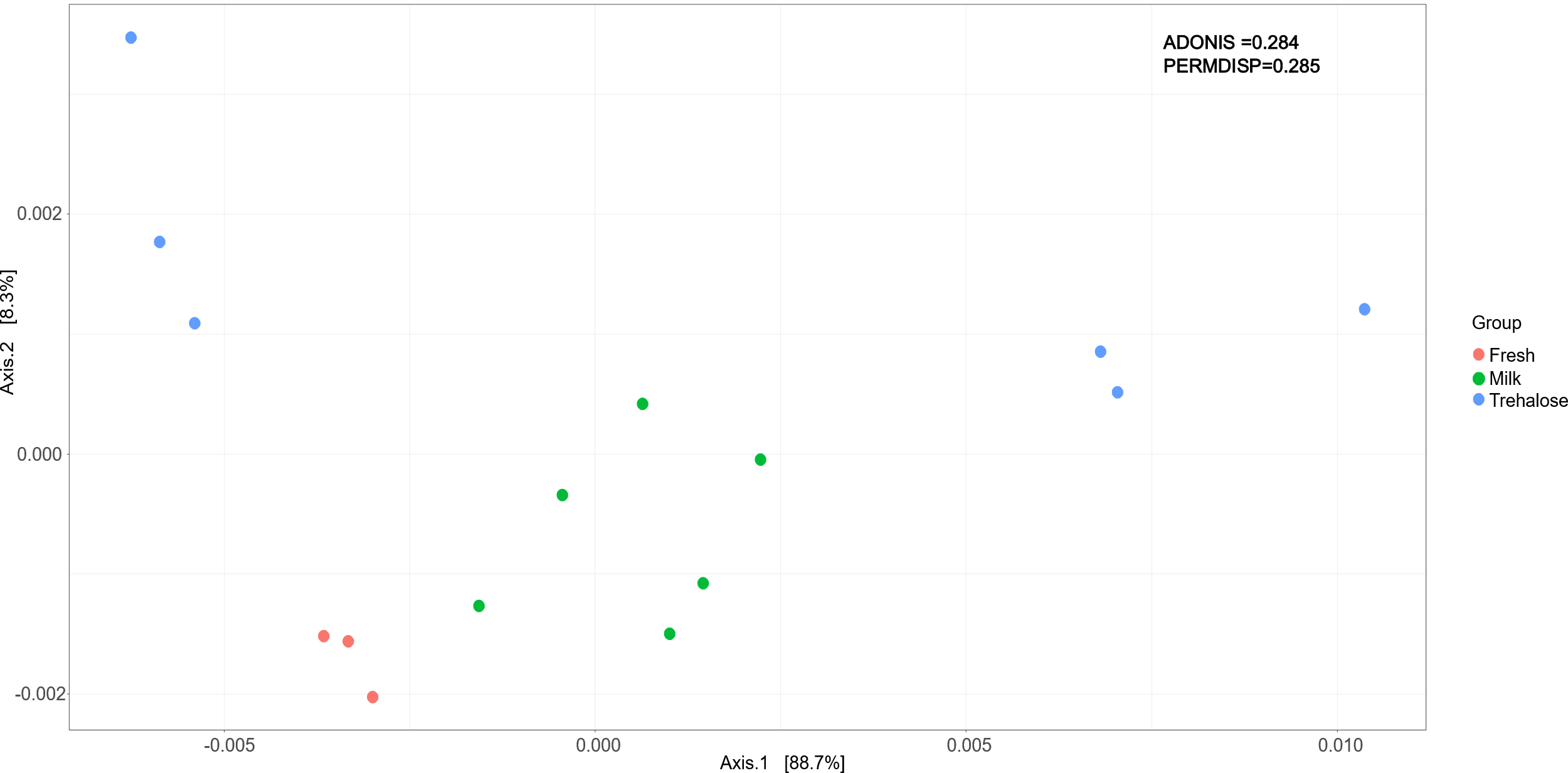


Supplemental Figure 2. Principle coordinate analysis (PCoA) of Bray Curtis dissimilarity matrix of microbial communities in kefir samples at fermentation completion as determined by shotgun metagenomic sequencing.
